# Danish general practitioners as gatekeepers for gynaecological patients in regions with different density of resident specialists in gynaecology: in which situations and to whom do they refer? A cross-sectional study

**DOI:** 10.1080/02813432.2023.2165085

**Published:** 2023-01-12

**Authors:** Alexander D. L. Laschke, Jan Blaakær, Charlotte Floridon Jensen, Mette Bach Larsen

**Affiliations:** a Speciallægeselskabet Alexander Laschke ApS, Aabenraa, Denmark; b Department of Clinical Research, University of Southern Denmark, Odense C, Denmark; c Department of Obstetrics and Gynaecology, Odense University Hospital, Odense C, Denmark; d Gynækologisk Klinik Holbæk, Holbæk, Denmark; e Department of Public Health Programmes, Randers Regional Hospital, University Research Clinic for Cancer Screening, Randers, Denmark

**Keywords:** Gatekeeping, referral and consultation, secondary care, gynaecology, health services accessibility, health equity

## Abstract

**Background:**

There are large differences in the density of Resident Specialists in Gynaecology (RSG) in the various regions of Denmark. It is unknown if this inequality affects the General Practitioner (GP) referral patterns of gynaecological patients.

**Objective:**

To investigate the GP referral patterns of gynaecological patients to the RSG or to the Hospital/Outpatient Clinic (HOC) in specific situations according to the regional density of RSGs. Moreover, to examine whether GPs prefer to refer to the HOC or to the RSG, or whether they were treated by the GP depending on the density of RSGs, specifically, in six benign gynaecological diagnoses.

**Design:**

A cross-sectional questionnaire survey.

**Setting:**

In Denmark, GPs serve as gatekeepers to secondary care, being responsible for referrals to resident specialists and in- and outpatient hospital care.

**Subjects:**

Five hundred Danish GPs were randomly selected and invited to take part in the questionnaire study. Main outcome measurements: Referral patterns: Own treatment, RSG, or HOC.

**Results:**

GPs prefer to refer their gynaecologic patients to RSGs rather than to HOCs. In addition, the study shows the higher the density of RSGs, the more gynaecological patients are referred to the RSG. This also applies to the six diagnoses examined.

**Conclusion:**

To allow patients’ equal access to specialist care, the density of RSGs must be equal all over the country.

## Introduction

In many European countries, the General Practitioner (GP) acts as a professional medical front line person between the wishes and needs of the population on the one hand and access to the specialised healthcare system on the other hand [1]. This gatekeeper system and GPs having a list of patients enrolled at their practice to ensure continuity of care has been seen as part of a comprehensive healthcare system and as a tool to ensure equal access for those in need of care [2,3].

In the course of a year, 86% of the Danish population comes into direct contact with their GP [4]. The composition of the population enrolled at the GPs list and those who actually contact the GP have an impact on the likelihood of referral to the various specialties [5]. Nevertheless, in Danish as well as in international studies, referral percentages are very similar, with 4–6% of GP contacts being referred to a resident specialist or to a Hospital/Outpatient Clinic (HOC) [6–9].

The GP referral patterns to resident specialists vary. A wide range of external conditions such as local access to resident specialist, social conditions and the general morbidity of those enrolled at the GP practice have been shown to have an impact on the proportion of patients that are referred [10]. Therefore, referrals occur for very different reasons and at different points in time during a patient contact. In addition, in Denmark there is an unequal distribution between health care regions of specialists, which might shift the referral pattern towards hospital care.

Within the gynaecological specialty, the GP can refer patients either to a HOC or to a Resident Specialist in Gynaecology (RSG).

It is unknown in which situations the GP refers gynaecological patients and, also, whether these patients are referred to an RSG or to the HOC. There is also a lack of knowledge as to whether the density of RSG influences the referral pattern; moreover, it is not known whether differences in the density of RSGs results in an inequality in the specialist treatment of gynaecological diseases.

The present study investigated the referral patterns for GPs referring gynaecological patients to the RSG or to the HOC in specific situations according to density of RSG. Further, we examined whether patients were referred to the HOC or to the RSG, or whether they were treated by the GP her/himself depending on the density of RSGs for six benign gynaecological diagnoses.

## Material and methods

### Setting

The Danish health care system is divided into five administrative regions which are defined geographically as the Capital Region (population ∼1.9 million), the Region of Zealand (∼0.8 million), the Southern Region (∼1.2 million), the Central Region (∼1.3 million), and the Northern Region (∼0.6 million). These regions govern primary and secondary health care services provided by GPs, hospitals, and resident specialists. GPs serve as gatekeepers to secondary care, including referrals to resident specialists and inpatient and outpatient hospital care. The Danish healthcare system is based on free and equal access to treatment and is mainly tax financed [3].

Each region politically decides how many resident specialists they require within each discipline, such as in gynaecology and obstetrics, but the number of female individuals per RSG varies considerably between regions, going from approximately 20,000 in the Capital Region of Denmark to approximately 145,000 in the North Denmark Region [11].

### Design

This was as a cross-sectional study based on questionnaire data from GPs.

### Study population

A total of 100 GPs were randomly selected from each of the five Danish regions with the help of a distribution key based on the total number of doctors in the respective region. Five hundred GPs were invited to take part in the questionnaire study.

### Questionnaires

The anonymised questionnaire comprised questions about demographic data of the GP, including age and sex. Furthermore, it asked in which situations the GP referred gynaecological patients and to whom (HOC or RSG). Six benign gynaecological diagnoses were provided as examples: (i) excessive and frequent menstruation with regular cycle, (ii) Lichen simplex chronicus, (iii) postmenopausal bleeding, (iv) menopausal and perimenopausal disorder, (v) dyspareunia, and (vi) insertion of (intrauterine) contraceptive device (IUD). The GP was asked which diagnoses (s)he treated her/himself or referred to a RSG or to the HOC.

The questionnaires were field tested before use. Three GPs were interviewed regarding their understanding of the questions and thereafter completed by five additional GPs. As the GPs deemed the questions understandable, no changes were made. For a list of questions, see Appendix Table A1.

### Data collection

The GPs received the questionnaire by postal mail in September 2020. A cover letter containing information on the study and a postage paid return envelope were enclosed with each questionnaire. The returned questionnaires were entered into Research Electronic Data Capture (REDCap) [12] by two independent persons and merged by a third person. Study data were collected and managed using REDCap hosted at the University of Southern Denmark.

### Data analysis

Characteristics of responding GPs were reported as numbers and proportions for each of the five regions. Differences between the responding GPs in each region were tested using Pearson’s Chi-Squared test.

Referral patterns of gynaecological patients from GPs overall and for six specific reasons were reported as numbers and proportions.

Associations between GPs reason for referring to RSG, HOC or both and density of RSG were calculated as odds ratios (OR) with 95% confidence intervals (CI) using generalized linear models for the binomial family. Likewise, the associations between GP referral to RSG, HOC or keeping patients in the GP’s practice, and density of RSG were calculated for the six specific diagnoses.

Data analyses were conducted using STATA statistical software 16 (StataCorp, College Station, TX, USA).

### Ethical approval

According to EU's General Data Protection Regulation (article 30), the project was listed at The Record of Processing Activities for Research Projects in Southern Denmark Region (j. no: 19/19630). According to the Consolidation Act on Research Ethics Review of Health Research Projects, Consolidation Act number 1083 of 15 September, 2017 section 14 (2) notification of questionnaire surveys or medical database research projects to the research ethics committee system is only required if the project involves human biological material. Therefore, this study was conducted without an approval from the committees (J.no.: S-20192000-78).

## Results

Of the 500 GPs who received a questionnaire, 347 GPs (69.4%) replied. Of these, 61.4% were female. Regarding age, 51.2% were younger than 50 years, and 76.3% were younger than 60 years. The majority (58.8%) had more than 10 years of professional experience as GPs and most commonly worked in practices with two to three doctors (45.2%). Most practices had both female and male GPs (52.3%). There were no statistically significant differences in any GP characteristics between regions (Table 1).

**Table 1.** Characteristics of responding general practitioners (*N* = 347).

	Capital Region *N* (%)	Region of Zealand *N* (%)	Southern Region *N* (%)	Central Region *N* (%)	Northern Region *N* (%)	All *N* (%)
Total	62 (17.9)	76 (21.9)	68 (19.6)	74 (21.3)	67 (19.3)	347 (100)
Sex						
Males	28 (45.2)	26 (34.2)	28 (41.2)	28 (37.8)	24 (35.8)	134 (38.6)
Females	34 (54.8)	50 (65.8)	40 (58.8)	46 (62.2)	43 (64.2)	213 (61.4)
Age						
≤50 yrs.	29 (47.5)	39 (51.3)	37 (54.4)	36 (48.7)	36 (53.7)	177 (51.2)
51–60 yrs.	17 (27.9)	18 (23.7)	18 (26.5)	20 (27.0)	14 (20.9)	87 (25.1)
≥61 yrs.	15 (24.6)	19 (25.0)	13 (19.1)	18 (24.3)	17 (25.4)	82 (23.7)
Years of experience as a GP						
5 yrs. or less	18 (29.0)	15 (19.7)	14 (20.6)	21 (28.4)	14 (20.9)	82 (23.6)
6–10 yrs.	6 (9.7)	12 (15.8)	18 (26.5)	12 (16.2)	13 (19.4)	61 (17.6)
More than 10 yrs.	38 (61.3)	49 (64.5)	36 (52.9)	41 (55.4)	40 (59.7)	204 (58.8)
Number of doctors in the practice						
One	23 (37.7)	24 (31.6)	21 (30.9)	21 (28.8)	22 (32.8)	111 (32.2)
2–3	28 (45.9)	39 (51.3)	30 (44.1)	30 (41.1)	29 (43.3)	156 (45.2)
4 or more	10 (16.4)	13 (17.1)	17 (25.0)	22 (30.1)	16 (23.9)	78 (22.6)
Distribution by sex						
All doctors are women	18 (29.5)	24 (31.6)	13 (19.1)	18 (24.3)	16 (23.8)	89 (25.7)
All doctors are men	16 (26.2)	15 (19.7)	15 (22.1)	16 (21.6)	14 (20.9)	76 (22.0)
Both female and male doctors	27 (44.3)	37 (48.7)	40 (58.8)	40 (54.1)	37 (55.2)	181 (52.3)
All doctors see gynaecological patients						
Yes	59 (96.7)	72 (94.7)	64 (95.5)	73 (98.7)	67 (100)	335 (97.1)

There were no statistically significant differences in any GP characteristics between regions (*p* > 0.05 for Pearson’s Chi-Squared test).

Capital Region: 20.000, Region of Zealand: 46.000, Southern Region: 52.000, Central Region: 71.000 and Northern Region: 145.000 women per Resident Specialist in Gynaecology.

### Referral patterns in specific situations

As shown in Table 2, 62.9% of GPs referred gynaecological patients to RSG and 9.6% to hospitals/outpatient clinics and 27.5% replied that they referred equally to both. In case of suspected malignancy or suspected severe illness, GPs referred mainly to the HOC. The majority of GPs prefer to refer their patients to RSG with regard to waiting time, patients’ wish, service and distance. In addition, 85.1% of GPs responded that they would prefer to refer patients to RSG, if waiting time and distance were the same as for HOCs.

**Table 2.** Numbers and proportions of General Practitioners (GPs) referring to Resident Specialist in Gynaecology (RSG), Hospital/Outpatient Clinic (HOC) and both equally in specific situation according to a survey of Danish GPs.

	Total		
	RSG	HOC	Both
	*N* (%)	*N* (%)	*N* (%)
Where do you most often refer to?	217 (62.9)	33 (9.6)	95 (27.5)
When do you choose one treatment site over another?			
Waiting time	164 (62.4)	88 (33.5)	N/A
Suspicion of cancer	37 (11.2)	282 (85.5)	11 (3.3)
The patients’ wish	150 (58.6)	49 (19.1)	57 (22.3)
Severity of the disease	32 (10.5)	262 (85.9)	N/A
Service	195 (82.6)	22 (9.3)	19 (8.1)
Distance	131 (66.5)	43 (21.8)	23 (11.7)
If waiting time and distance were the same; where would you prefer to refer?	239 (85.1)	42 (15.0)	–

GP: General Practitioner; RSG: Resident Specialist in Gynaecology; HOC: Hospital/Outpatient Clinic; N/A: not available, missing or insufficient data.

Table 3 shows, that in regions with a lower density of RSGs than the highest, GPs less frequently referred patients to the RSG. In relation to waiting time and distance, as the density of RSG decreased, the probability of being referred to hospital increased.

**Table 3.** General Practitioners’ reason for referring gynaecological patients to a Resident Specialist in Gynaecology, to a hospital/outpatient clinic or both.

	RSG OR (95% CI)	HOC OR (95% CI)	Both OR (95% CI)
Most often referred to			
Capital Region	1 (Ref)	1 (Ref)	1 (Ref)
Region of Zealand	0.31* (0.10; 0.98)	0.81 (0.05; 13.27)	6.19* (1.34; 28.59)
Southern Region	0.15* (0.05; 0.48)	3.81 (0.41; 35.07)	9.23* (2.03; 42.04)
Central Region	0.04* (0.01; 0.11)	8.45* (1.04; 68.65)	33.43* (7.60; 146.98)
Northern Region	0.04* (0.01; 0.12)	22.41* (2.89; 173.80)	17.86* (4.01; 79.49)
Waiting time			
Capital Region	1 (Ref)	1 (Ref)	1 (Ref)
Region of Zealand	0.44* (0.22; 0.91)	4.06* (1.10; 14.96)	1.81 (0.32; 10.19)
Southern Region	0.34* (0.16; 0.70)	8.19* (2.30; 29.23)	0.49 (0.04; 5.48)
Central Region	0.15* (0.07; 0.32)	11.97* (3.42; 41.84)	1.86 (0.33; 10.48)
Northern Region	0.22* (0.11; 0.47)	10.98* (3.10; 38.81)	N/A
Suspicion of cancer			
Capital Region	1 (Ref)	1 (Ref)	1 (Ref)
Region of Zealand	0.65 (0.28; 1.49)	1.26 (0.61; 2.59)	3.39 (0.37; 31.12)
Southern Region	0.30* (0.11; 0.84)	2.17 (0.97; 4.83)	4.84 (0.55; 42.62)
Central Region	0.04* (0.01; 0.34)	8.96* (2.88; 27.93)	N/A
Northern Region	0.10* (0.02; 0.44)	6.35* (2.22; 18.19)	0.92 (0.06; 15.09)
The patients’ wish			
Capital Region	1 (Ref)	1 (Ref)	1 (Ref)
Region of Zealand	0.97 (0.50; 1.90)	1.02 (0.38; 2.77)	3.04* (1.05; 8.84)
Southern Region	0.85 (0.42; 1.69)	1.60 (0.61; 4.16)	2.69 (0.90; 8.06)
Central Region	0.65 (0.33; 1.30)	0.82 (0.29; 2.32)	2.43 (0.81; 7.25)
Northern Region	0.92 (0.46; 1.85)	1.18 (0.44; 3.22)	2.00 (0.64; 6.22)
Severity of the disease			
Capital Region	1 (Ref)	1 (Ref)	1 (Ref)
Region of Zealand	1.06 (0.37; 3.02)	1.42 (0.66; 3.06)	0.40 (0.04; 4.52)
Southern Region	1.86 (0.69; 5.01)	0.62 (0.30; 1.29)	1.88 (0.33; 10.61)
Central Region	N/A	1.92 (0.85; 4.32)	0.83 (0.11; 6.09)
Northern Region	0.37 (0.09; 1.49)	2.33 (0.98; 5.55)	0.92 (0.13; 6.76)
Service			
Capital Region	1 (Ref)	1 (Ref)	1 (Ref)
Region of Zealand	0.96 (0.48; 1.91)	0.40 (0.04; 4.52)	1.23 (0.20; 7.62)
Southern Region	1.08 (0.53; 2.21)	0.45 (0.04; 5.06)	0.91 (0.12; 6.66)
Central Region	0.53 (0.27; 1.05)	2.17 (0.41; 11.62)	3.13 (0.63; 15.67)
Northern Region	0.48* (0.24; 0.97)	7.22* (1.56; 33.47)	2.42 (0.45; 12.95)
Distance			
Capital Region	1 (Ref)	1 (Ref)	1 (Ref)
Region of Zealand	0.96 (0.49; 1.89)	1.65 (0.15; 18.61)	0.81 (0.11; 5.93)
Southern Region	0.54 (0.27; 1.09)	7.00 (0.84; 58.59)	1.88 (0.33; 10.61)
Central Region	0.19* (0.09; 0.41)	13.00* (1.65; 102.43)	3.64 (0.74; 17.80)
Northern Region	0.24* (0.11; 0.51)	25.96* (3.36; 200.35)	3.50 (0.70; 17.54)

Data presented as odds ratio (OR) with 95% confidence interval (CI).

Capital Region: 20.000, Region of Zealand: 46.000, Southern Region: 52.000, Central Region: 71.000 and Northern Region: 145.000 women per Resident Specialist in Gynaecology.

GP: General Practitioner; RSG: Resident Specialist in Gynaecology; HOC: Hospital/Outpatient Clinic; *Significant Result; N/A: not available, missing or insufficient data.

### Referral patterns according to diagnosis

As can be seen from Table 4, with regard to the six benign gynaecological diagnoses, GPs were more likely to refer to the RSG than to the HOC and more likely to carry out the treatment themselves than to refer patients to the HOC in all other diagnoses than Postmenopausal bleeding. Apart from the diagnoses of Menopausal and perimenopausal disorders and the Insertion of IUD, the general practitioners were more likely to refer patients to RSG than to perform the treatment themselves.

**Table 4.** Referral pattern of GPs analysed by diagnosis.

	RSG	HOC	GP
	Total	Total	Total
	*N* (%)	*N* (%)	*N* (%)
Excessive and frequent menstruation with regular cycle	203 (58.5)	56 (16.1)	184 (53.0)
Lichen simplex chronicus	263 (75.8)	51 (14.7)	88 (25.4)
Postmenopausal bleeding	174 (50.1)	162 (46.7)	38 (11.0)
Menopausal and perimenopausal disorder	149 (42.9)	N/A	272 (78.4)
Dyspareunia	248 (71.5)	74 (21.3)	82 (23.6)
Insertion of IUD	145 (41.8)	N/A	258 (74.3)

GP: General Practitioner; RSG: Resident Specialist in Gynaecology; HOC: Hospital/Outpatient Clinic; N/A: not available, missing or insufficient data.

Table 5 demonstrates, for the six benign gynaecological diagnoses, that GPs in the region with the lowest density of RSGs (Northern Region) referred to a RSG to a lesser extent than in the region with the highest density (Capital Region). On closer inspection of the table shows, this difference was significant for Excessive and frequent menstruation with regular cycle, Lichen simplex chronicus, Postmenopausal bleeding, Dyspareunia and Insertion of IUD. Insertion of IUD was more often treated by the GPs themselves in regions where the density of RSG was not the highest. The same applied to patients with Lichen simplex chronicus, although these patients were also referred to the HOC more frequently in regions with a lower density of RSG.

**Table 5.** Table showing proportions of GPs who report to refer to (i) Resident Specialist in Gynaecology, (ii) Hospital/Outpatient Clinic or (iii) provide own treatment of five specific conditions divided by region.

	RSG OR (95% CI)	HOC OR (95% CI)	GP OR (95% CI)
Excessive and frequent menstruation with regular cycle			
Capital Region	1 (Ref)	1 (Ref)	1 (Ref)
Region of Zealand	0.93 (0.46; 1.88)	0.06 (0.01; 0.28)	1.62 (0.82; 3.20)
Southern Region	0.83 (0.40; 1.70)	0.67 (0.31; 1.45)	0.74 (0.37; 1.49)
Central Region	0.67 (0.33; 1.35)	0.81 (0.39; 1.70)	1.18 (0.60; 2.31)
Northern Region	0.39 (0.19; 0.80)	N/A	1.23 (0.62; 2.47)
Lichen simplex chronicus			
Capital Region	1 (Ref)	1 (Ref)	1 (Ref)
Region of Zealand	0.80 (0.22; 2.99)	0.40 (0.04; 4.52)	2.90 (1.07; 7.83)
Southern Region	0.29 (0.09; 0.95)	4.58 (0.95; 22.08)	1.61 (0.55; 4.72)
Central Region	0.36 (0.11; 1.17)	4.69 (0.99; 22.27)	3.46 (1.29; 9.26)
Northern Region	0.03 (0.01; 0.08)	22.89 (5.16; 101.53)	9.62 (3.65; 25.33)
Postmenopausal bleeding			
Capital Region	1 (Ref)	1 (Ref)	1 (Ref)
Region of Zealand	0.31 (0.14; 0.71)	4.09 (1.63; 10.23)	1.10 (0.43; 2.82)
Southern Region	0.24 (0.11; 0.56)	5.84 (2.32; 14.69)	0.79 (0.28; 2.18)
Central Region	0.05 (0.02; 0.13)	21.21 (8.29; 54.26)	0.52 (0.17; 1.55)
Northern Region	0.09 (0.04; 0.21)	17.21 (6.72; 44.09)	0.28 (0.07; 1.07)
Menopausal and perimenopausal disorder			
Capital Region	1 (Ref)	1 (Ref)	1 (Ref)
Region of Zealand	2.13 (1.07; 4.24)	N/A	0.59 (0.24; 1.43)
Southern Region	1.52 (0.75; 3.09)	0.32 (0.03; 3.14)	0.51 (0.21; 1.25)
Central Region	1.46 (0.73; 2.93)	1.22 (0.26; 5.66)	0.57 (0.23; 1.39)
Northern Region	0.89 (0.43; 1.84)	N/A	0.59 (0.24; 1.46)
Dyspareunia			
Capital Region	1 (Ref)	1 (Ref)	1 (Ref)
Region of Zealand	1.05 (0.43; 2.53)	5.23 (0.61; 44.63)	0.98 (0.47; 2.04)
Southern Region	0.76 (0.32; 1.82)	11.77 (1.47; 94.06)	0.48 (0.21; 1.11)
Central Region	0.51 (0.22; 1.16)	25.81 (3.36; 197.96)	0.67 (0.31; 1.45)
Northern Region	0.16 (0.07; 0.37)	62.85 (8.23; 479.83)	0.44 (0.19; 1.03)
Insertion of IUD			
Capital Region	1 (Ref)	1 (Ref)	1 (Ref)
Region of Zealand	0.81 (0.41; 1.59)	0.17 (0.02; 1.45)	2.19 (1.04; 4.61)
Southern Region	0.79 (0.40; 1.57)	0.38 (0.07; 2.01)	1.41 (0.68; 2.92)
Central Region	0.72 (0.37; 1.42)	0.52 (0.12; 2.28)	2.96 (1.35; 6.51)
Northern Region	0.40 (0.19; 0.82)	N/A	2.39 (1.10; 5.21)

GP: General practitioner; RSG: Resident Specialist in Gynaecology; HOC: Hospital/Outpatient Clinic.

Capital Region: 20.000, Region of Zealand: 46.000, Southern Region: 52.000, Central Region: 71.000 and Northern Region: 145.000 women per Resident Specialist in Gynaecology.

N/A: not available, missing or insufficient data.

## Discussion

### Statement of principal findings

This cross-sectional study showed that the referral patterns of GPs was highly dependent on the density of RSGs. The higher the density of RSGs, the more likely that gynaecological patients were referred to the RSG, and conversely, the lower the density of RSGs, the more likely that gynaecological patients were referred to the HOC. GPs most often refer their gynaecological patients to the HOC in cases of suspicion of cancer or other severe disease.

### Strengths and weaknesses of the study

Because none of the previously existing questionnaires we could find on this topic addressed all the items we wanted to include in this study, we developed a study-specific questionnaire. This ensured that the relevant questions were included and that the context was given.

We used paper questionnaires as it was not possible obtain a list of email addresses of the GPs due to the General Data Protection Regulation (GDPR). Paper questionnaires have shown declining response rates over the past decade. A low response rate may induce selection bias because respondents may differ systematically from non-respondents, and the study population will thus not represent the target population [13]. However, we achieved a fair response rate with a percentage of 69.4% and with 61.4% females compared to the Danish national average of 58.1% [14]. Thus, the risk of selection bias must be considered low. However, because we did not have access to any information on the targeted study sample, we could not perform a responder – non-responder analysis.

For logistic reasons, we selected and invited 100 GPs from each Danish region. This corresponds to 15% of all GPs in Denmark. However, as the number of GPs in the different regions is not the same in absolute numbers, this resulted in a different percentage of invitations between regions, ranging from 9.7% (Capital Region) to 35.1% (Northern Region). Since GPs in Denmark, regardless of the region in which they practice, have the same education at the respective time in their career and the distribution of GPs in the regions is almost the same with regard to sex and age [14], we believe that this study sample is generalisable to the GP population in its entirety. The fact that we found no differences in GP characteristics over the regions strengthens the credibility of our results.

The present study has been carried out in Denmark under Danish conditions in the health system. However, the results should be comparable with health systems that are similarly structured e.g. GP as gatekeeper, especially with the other Scandinavian countries thus we assume that the conditions would be similar due to the great cultural proximity.

### Findings in relation to other studies

Women with gynaecological problems, who are referred to an RSG are always examined by a specialist but when referred to an HOC, they would often be examined by a doctor, who is not yet a specialist but still in training. To compensate for this, HOCs are organized such that doctors in training can always call in a specialist [15], although this depends on whether the examining doctor decides to call a specialist or not. Due to lack of experience, it may happen that the doctor in training comes to misjudgements and does not call a specialist although it would be indicated. Thus, this may delay the correct diagnosis of a serious disease [16]. This difference means that unless all patients have equal access to relevant care, there would be an inequality in the quality of care depending on in which part of the country they live, which, in turn, can have an impact on the health of this group of the population.

Our study demonstrated that GPs prefer to refer their gynaecological patients to RSG; only 9.6% of GPs refer their patients exclusively to the hospital, although most would refer their gynaecological patients directly to the hospital, if they suspect cancer or another severe diagnosis. We examined five geographic regions with different densities of RSG and found that the referral pattern depends on the density of RSG. These results are in agreement with previous studies that have shown that if the number of resident specialists increases, more patients are referred to a resident specialist and at the same time fewer patients are referred to hospitals [17].

With regard to the diagnoses examined, the present study shows that the referral pattern is strongly dependent on the density of RSG in the local region and for five of the six gynaecological diagnoses examined, there was a significantly lower chance for the patient to be referred to an RSG in the region with the lowest density compared to the region with the highest density of RSG.

The national average distance from the patient’s place of residence to the hospital is greater than the average distance from the patient’s place of residence to the RSG in the region with the highest density of RSG. This results in a longer transport time and more costs for the patients who live in the region with lowest density of RSG. This can have detrimental effects, as it has shown in previous studies that there is an association between travel distance and cancer prognosis [18,19]. We also know that the distance to the hospital is linked to an increasing diagnostic interval for cancer [20]. As far as we know, this has not been investigated in relation to the density of RSGs. However, since the RSG is a specialist, it is not unlikely that such studies would obtain similar results. When delays are discussed in the diagnosis of cancer, for example, patient delays, GP delays, and system delays are mentioned [21], but the density of resident specialists has not been taken into account, although it is known that increased availability of specialist care translates into higher referral rates [22].

### Possible mechanisms and implications for clinicians or policy makers

In regions with a lower density of resident specialists in gynaecology, women are less frequently referred to a resident specialist in gynaecology. If there are regions in the same country with different densities of resident specialists in gynaecology, one must assume that the population will have an unequal opportunity to have a specialist examination. This results in an injustice in the healthcare system within the same country. Whether or not this inequality should be accepted or not is a political decision, but our results indicate that there are significant differences between regions that may have an impact on the gynaecologic treatment of women.

Clearly, further studies are needed to determine the exact consequences of the difference in referral patterns in terms of treatment outcomes. However, the results from our study should already facilitate the future planning of health care in gynaecology with the aim of reducing inequality in the access to RSG.

## Appendix

**Table A1.** Questionnaire.

These questions are about you and your practice.	
In which region is the practice you work in?	North Denmark Region
	Central Denmark Region
	Region of Southern Denmark
	Region Zealand
	Capital Region of Denmark
How long have you been a general practitioner?	Less than 2 yrs.
	2–5 yrs.
	6–10 yrs.
	More than 10 yrs.
What is your SEX?	Man
	Woman
How old are you?	Younger than 40 yrs.
	40–45 yrs.
	46–50 yrs.
	51–55 yrs.
	56–60 yrs.
	61–65 yrs.
	66–70 yrs.
	71 or older
How many doctors work in the practice you work in?	I'm the only doctor
	We are 2–3 doctors
	We are 4 doctors or more
How is the sex distribution in your practice?	All doctors are women
	All doctors are men
	Both female and male doctors
Do all doctors in your practice see gynaecological patients?	Yes
	No
	Don’t know
These questions are about where you prefer to refer your gynaecological patients.	
When you refer a woman to a gynaecologist, where do you most often refer to?	Hospital/Outpatient Clinic Resident Specialist in Gynaecology
	Both equally
If there were the same conditions in terms of waiting time and distance, where would you prefer to refer to?	Hospital/Outpatient Clinic Resident Specialist in Gynaecology
If there was a Resident Specialist in Gynaecology closer to your practice, would you refer more women to the Resident Specialist in Gynaecology instead of the Hospital/Outpatient Clinic?	Yes No

When do you choose one treatment site over the other?		
	Hospital/Outpatient Clinic	Resident Specialist in Gynaecology
Waiting time	O	O
Suspicion of cancer	O	O
Patients wish	O	O
Severity of the disease	O	O
Service	O	O
Distance	O	O

What do you mostly do with women for the diagnoses listed below?			
	Referral to Hospital/Outpatient Clinic	Referral to Resident Specialist in Gynaecology	Treat in my own practice
DN920B, menorraghia et polymenorrhoea	O	O	O
DL280A, Lichen sclerosus et atrophicus	O	O	O
DN950, metrorrhagia menopausalis	O	O	O
DN959, menopausal and perimenopausal disorder	O	O	O
DN941, dyspareunia	O	O	O
DZ301, insertion of IUD	O	O	O
